# Nudging to donate organs: do what you like or like what we do?

**DOI:** 10.1007/s11019-021-10007-6

**Published:** 2021-03-17

**Authors:** Sergio Beraldo, Jurgis Karpus

**Affiliations:** 1 Department of Economics and Statistics, University of Napoli, Naples, Italy; 2 Faculty of Philosophy, Philosophy of Science and the Study of Religion, LMU Munich, Munich, Germany; 3 Faculty of Psychology and Educational Sciences, General and Experimental Psychology, LMU Munich, Munich, Germany

**Keywords:** Default rule nudge, Automatic enrolment, Organ donation, As judged by themselves, Mandated active choice

## Abstract

An effective method to increase the number of potential cadaveric organ donors is to make people donors by default with the option to opt out. This non-coercive public policy tool to influence people’s choices is often justified on the basis of the *as-judged-by-themselves* principle: people are nudged into choosing what they themselves truly want. We review three often hypothesized reasons for why defaults work and argue that the *as-judged-by-themselves* principle may hold only in two of these cases. We specify further conditions for when the principle can hold in these cases and show that whether those conditions are met is often unclear. We recommend ways to expand nationwide surveys to identify the actual reasons for why defaults work and discuss mandated choice policy as a viable solution to many arising conundrums.

## Introduction

The number of potential cadaveric organ donors in a given country largely depends on how people’s options to become and not to become a potential donor are framed. It also depends on how and when people choose between them. A mandated choice policy requires everyone to decide on whether they wish to be considered as a potential donor or not. Each person is free to choose what they want, but they must actively do it at some point during their life. A voluntary choice policy may encourage active choosing but does not force it. Most countries have implemented some version of the latter.^1^ Since expressing one’s choice is voluntary, this requires taking a stance on how to treat the organs of deceased persons who never made their choice known (or indeed perhaps never thought about the matter at all). An opt-in system assumes that someone is not a potential donor unless that person actively registers as such, for example, by obtaining a donor registration card. An opt-out system assumes that people are donors by default unless at some point during their lives they explicitly state otherwise. What is set to be the default choice matters. It is often the case that most people in opt-in systems do not register as donors and most people in opt-out systems do not express their wish to be non-donors.

This does not mean that people in opt-out systems are more willing to donate their organs than those in opt-in systems. While one study inferred such a link from survey data spanning 15 European Union member states in the early 2000s (Mossialos et al. 2008), the more recent Special Eurobarometer 333a report suggests no clear-cut difference in people’s willingness to donate organs between countries that have adopted one versus the other framework (European Commission 2010). In general, most people do not express a decision—either in favour or against donation—regardless of the system in place. Surveys conducted over the past few decades suggest that most people in Europe do not register a decision (Rosenblum et al. 2012), do not hold a donor registration card (European Commission 2007), and do not discuss the issue of organ donation with their family (European Commission 2010).

The fact that many people in opt-out countries do not express a refusal to donate also doesn’t mean that they *proactively choose* to become potential donors. A recent systematic review shows that people in opt-out countries tend to be less aware of the consent system in place than those in opt-in countries, suggesting that this is at least partially attributable to ignorance (Molina-Pérez et al. 2019). Be that as it may, a switch from an opt-in (explicit consent) to an opt-out (presumed consent) system can significantly increase the number of *potential* donors in a jurisdiction in which the switch is enacted (Johnson and Goldstein 2003). Whether that in itself can also increase *actual* posthumous donation rates is debated. Arshad et al. (2019) recently compared opt-in and opt-out systems across 35 countries and found no significant difference in these rates. In a systematic review conducted a decade earlier, Rithalia et al. (2009) found that, although actual donation rates tended to be higher in opt-out countries, there was not enough evidence to attribute that to the consent system in place. In any case, many countries have recently switched or are considering a switch from an opt-in to an opt-out system with the hope that a greater number of potential donors would ultimately result in a greater number of actual transplantations and, with that, prolonged lives.

Matching the supply and demand between those willing to donate and those in need of an organ is something we should urgently strive for. However, whether changing what is set to be people’s default choice is (i) the most effective and (ii) a legitimate method to attain this goal is debated (English and Wright 2007; Rithalia et al. 2009; MacKay and Robinson 2016; Arshad et al. 2019; Prabhu 2019). While (i) is a contested empirical matter, (ii) depends on the basis on which the default rule method is ultimately justified. We will focus on a popular justification taken from the behavioural science of nudge: the *as-judged-by-themselves* principle. According to this principle, the default choice aligns people’s actual choices with what they themselves truly want to do. This is certainly not the only and most likely also not the main justification for enacting a switch. However, it is one that allows the switch from an opt-in to an opt-out system to be sanctioned without necessarily needing as detailed a public debate—for example, in asking people to actively engage with reasons as to why their choices should change—as other justifications of such a policy may require. After all, if a policy can simultaneously meet the personal interests of those who are waiting for a transplant and those who are in the position to supply one, it is an obvious win–win for all.

A strong case for sanctioning the switch of the default choice can be built by inferring people’s wishes from nationwide surveys. Irrespective of the system in place, majorities of respondents often answer affirmatively when asked whether they would be willing to donate their organs after death. For example, according to the special consultation launched by the European Commission in 2009, 55% of respondents from the 27 European Union member states were willing to donate (27% were not and 18% were unsure; European Commission 2010, p. 15). This suggests a dichotomy between people’s stated and revealed preferences, i.e., between what people *declare* to prefer in a survey and what they *reveal* to prefer through their actual choices. For example, in Germany, which currently operates under the opt-in system, a recent survey found that while 84% of Germans had a positive attitude toward organ donation, only 36% owned an organ donor card (BZgA 2019, pp. 17, 104). This mismatch between people’s stated preferences (willingness to donate) and their actual choices (failure to register as potential donors) gives rise to a policy recommendation to nudge people into becoming potential donors, and changing people’s default choice is a particularly effective way to do so.

In this paper we argue that, in the context of organ donation, whether people are nudged into choosing what they themselves deem to be their best choice crucially depends on what ultimately causes them to stick with the default option. Conditional on the actual cause, different conclusions will emerge regarding the type of justification and, with that, the extent of public engagement needed when enacting a nudge-based intervention.

Three often discussed causes of the effectiveness of defaults are: (i) people’s tendency to procrastinate and the status quo bias, (ii) reference-point-dependent preferences and loss aversion, and (iii) people’s perception of the default option as an implicit recommendation of policy-makers as experts in the decision problem at hand (Kahneman et al. 1991; Camerer et al. 2003; Johnson and Goldstein 2003; McKenzie et al. 2006; Halpern et al. 2007; Davidai et al. 2012; Sunstein and Reisch 2013). We will argue that the *as-judged-by-themselves* principle may be used to justify a switch of the default choice only in cases (i) and (ii), but not (iii). We will specify further assumptions that have to be made for the principle to be met in cases (i) and (ii). We will then suggest a number of reasons for why there may be a mismatch between people’s stated preferences and their actual choices, suggest ways to expand nationwide surveys in order to identify the actual causes of the default rule’s effectiveness, and discuss mandated choice policy as a viable solution to many arising conundrums.

## Nudge and the as-judged-by-themselves principle

Since Thaler and Sunstein’s seminal book *Nudge* (2008), it is customary to refer to the opt-in (explicit consent) and the opt-out (presumed consent) systems as a matter of a country’s adopted choice architecture. Put simply, choice architecture specifies conditions in which choices are made. These include, for example, the way choice options are presented, the extent of information provided to decision-makers, and whether some particular option is set as one’s default choice in case of inaction. From choosing between taking the stairs or the escalator to deciding how much to save for one’s retirement, choice architecture can have a significant impact on what people do in many walks of life (Thaler and Sunstein 2008; Sunstein 2016).

Thaler and Sunstein’s starting point, as that of most proponents’ of nudge-based policy interventions (e.g., Camerer et al. 2003), is the claim that people often make imperfect decisions due to limitations of human cognitive abilities. Decision-makers who always act in line with their true rational preferences are non-existent *econs* or *homines economici*. Only *econs* are able to “think like Albert Einstein, store as much memory as IBM’s Big Blue, and exercise the willpower of Mahatma Gandhi” (2008, p. 6). If assessed against the backdrop of the choices an *econ* would make, decisions made by real people often appear unsatisfactory: these decisions would be different if people “possessed complete information, unlimited cognitive abilities, and complete self-control” (2008, p. 5).

For these reasons, it may be desirable to steer people’s decisions toward those that their own ‘better selves’ would make. As Thaler and Sunstein put it, “So long as people are not choosing perfectly, some changes in the choice architecture could make their lives go better (as judged by their own preferences, not those of some bureaucrat)” (2008, p. 10). The point that it is people’s own judgement about what is best for them that nudge-based policy interventions are meant to tap into, is reiterated more recently by Thaler: “We just want to reduce what people would themselves call errors” (2015, p. 326).

Since nudge-based policy interventions merely change conditions in which choices are made without taking any of the available options away from decision-makers, this allegedly non-intrusive form of paternalism leaves people free to do what they like and to opt out of undesirable arrangements if they indeed want to do so. Crucially, the success of a nudge in improving people’s decision-making is assessed from the choosers’ perspective by determining how well the policy aligns people’s choices with what they themselves want. This is the core of the *as-judged-by-themselves* principle, the purpose of which is to “discipline the content of paternalistic interventions” (Sunstein 2018, p. 3). Importantly also, the principle is concerned with personal interests of individuals and not the interests of groups, unless, of course, everyone’s personal interests are perfectly aligned with the interests of the society at large (however the latter are defined).

Ambiguities can arise, however, in determining what people’s ‘better selves’ would in fact choose, for this requires purifying people’s preferences from what they themselves would call errors to find what their ‘inner rational selves’ truly prefer (Hausman 2012; Infante et al. 2016). We identify three necessary requirements concerning latent (purified) preferences that people may have but not always act upon, in order for the *as-judged-by-themselves* principle to justify a nudge-based policy intervention.

The first and seemingly trivial requirement is that these preferences exist. A nudge can align people’s choices with what they themselves truly want only if they indeed truly want it. Moreover, people must strictly prefer the option which they are nudged into choosing to the option which they choose in the absence of the nudge and this preference should be sufficiently stable.^2^ This precludes preferential indifference and cases where one’s true preference continually fluctuates. Why must this be so? First, if people were truly preferentially indifferent between the choice options in question, we could not justify a nudge-based policy intervention on the basis that, compared to the status quo, it *improves* the alignment of people’s actual choices with what they themselves truly want. Second, if people’s true preferences were unstable, the success of a nudge in aligning people’s choices with what they truly prefer would be fleeting as well. Thus, in order for the *as-judged-by-themselves* principle to justify a nudge-based policy intervention, people have to have a strict and sufficiently stable preference over the options in question. (A sufficiently stable preference could mean that people prefer the option which they are nudged into choosing most of the time.) Call this requirement R1.

The second requirement (R2) is that the latent (purified) preferences are all-things-considered subjective evaluative judgements that encapsulate all factors that decision-makers themselves deem to be important and motivationally relevant for making their choice. Put differently, these preferences are people’s *total*, not merely *partial*, evaluative rankings of considered options (Hausman 2005, 2012). For an example consider Alice who prefers to donate her organs after death because of her desire to save lives. She also prefers not to donate organs because of a religious belief that she holds. Thus, Alice’s two *partial* evaluative rankings of her options pull her in opposite directions—one towards becoming a potential donor and the other towards not becoming one. In the end, Alice needs to make a trade-off between two competing values that she herself cares about. As such, in order to assess whether a nudge could help align Alice’s actual choice with what she herself truly wants, we need to know the trade-off that Alice herself is inclined to make. In other words, we need to know her own true *total* evaluative ranking of the options in question.

The third requirement (R3) is that the preferences in question are antecedent to the introduction of a nudge. The *as-judged-by-themselves* principle can justify a nudge-based intervention only if the nudge aligns people’s choices with what they already prefer, not with what they would prefer under different circumstances. For example, the principle cannot be used to justify an intervention if that intervention itself shapes what people prefer. Although in such case people’s choices would indeed be aligned with what they prefer post the intervention, it would be incorrect to say that the intervention better aligns people’s choices with what they themselves truly want compared to what was the case prior to the intervention (for a further discussion of this point see Sugden 2017, 2018 and Sunstein 2018).

## Do what you like or like what we do?

We now turn to the specific issues at hand in the context of organ donation by means of a simple formal analysis. Our aim is to assess the most common explanations of the effectiveness of defaults in this setting. In particular, we wish to examine whether these explanations allow justifying default rule nudge interventions on the basis of the *as-judged-by-themselves* principle.

Let D and ND denote the options of becoming and not becoming a potential organ donor respectively. We follow standard notation in using ≻ and ∼ to denote a decision-maker’s strict preference and preferential indifference over these options. We use subscripts ‘t’, ‘s’, and ‘r’ to distinguish between people’s true, stated, and revealed preferences.

By true preference we mean a latent (rational) preference that a decision-maker has but sometimes fails to act upon. In light of our discussion earlier, D ≻_t_ ND means that the decision-maker’s ‘better’ inner rational self strictly prefers becoming a potential organ donor to not becoming one, ND ≻_t_ D means that her ‘better’ self strictly prefers not becoming a potential donor, and D ∼_t_ ND means that her ‘better’ self is truly indifferent between becoming and not becoming a donor. A nudge-based policy intervention aims to align the decision-maker’s actual choice with this true preference. We also use D ⊥_t_ ND to denote a case where the decision-maker’s true preference is incomplete: her ‘better’ inner rational self neither strictly prefers one option to the other, nor is she preferentially indifferent between the two. In other words, the decision-maker does not have a fully formed latent preference over D and ND. (Differently from the orthodox decision theory, we allow rational decision-makers to have incomplete preferences. However, treating this as a case of irrationality does not change our conclusions.)

By stated preference we refer to what the decision-maker declares to prefer verbally or in writing. For example, we write D ≻_s_ ND for someone who answers affirmatively when asked in a nationwide survey whether she would be willing to donate her organs after her death.

Lastly, by revealed preference we refer to the decision-maker’s actual choice. For example, if under the opt-in system the decision-maker is not a potential donor (in other words, she does not choose to become a donor), we write ND ≻_r (opt-in)_ D. We assume that revealed preference is always strict (though relaxing this assumption does not have a bearing on our analysis).

For our discussion going forward, we assume two stylized facts:

1. People’s stated preferences are to become potential organ donors: D ≻_s_ ND.
2. A default rule nudge is effective in the sense that the default option determines what people do: ND ≻_r (opt-in)_ D and D ≻_r (opt-out)_ ND.

Both assumptions are strong and at face value they are clearly false. It is not the case that everyone in any population expresses their willingness (and nor is everyone in fact willing) to donate their organs after death. Similarly, while a default rule nudge may influence many people’s choices, it certainly does not work on everyone. For our analysis, it is sufficient to assume that the above statements hold for the majority of people. This of course completely sidesteps the crucially important question of whether it is permissible to nudge an entire population into choosing something that a minority of people do not in fact prefer (Bovens 2009). We will not address this question here. An alternative approach is to follow our analysis with a single individual in mind for whom the above two statements hold. In that case our goal is to examine whether a default rule nudge intervention is justified on the basis of the *as-judged-by-themselves* principle for that individual alone.

Setting these important matters aside and accepting the two stylized facts going forward, in an opt-out system people’s stated and revealed preferences agree, but in an opt-in system they don’t. This in itself does not suffice to justify a default rule nudge policy intervention on grounds that it meets the *as-judged-by-themselves* principle. For that we need to make a reasoned assumption about what people’s true preferences are. If there are good grounds to believe that people’s true preferences coincide with their stated preferences, the principle *can* justify such intervention. If there are good grounds to believe that they don’t, it *can’t*. We thus need to establish what assumptions about people’s true preferences are accorded by psychological mechanisms that are postulated to underlie the effectiveness of defaults in influencing people’s actual choices.

### The first hypothesis (H1)

One popular explanation for why default rule nudges work is the status quo bias in people’s actual choices that emerges due to people’s tendency to procrastinate (Camerer et al. 2003; Johnson and Goldstein 2003; Halpern et al. 2007; Davidai et al. 2012; Sunstein and Reisch 2013). If enacting a decision to abandon the status quo option requires one to exert effort (for example, in obtaining and filling out a form, writing and sending an e-mail, making a phone call) it can be tempting to postpone this act till a more convenient time in the future. If people continually do that, whatever is set to be their status quo option will have a significant impact on their actual choice. This allows in principle to infer from people’s stated preferences that they truly prefer being potential organ donors (D ≻_s_ ND → D ≻_t_ ND) and to assume that in an opt-in system they unceasingly defer aligning their actual choices with what they truly prefer (ND ≻_r (opt-in)_ D despite D ≻_t_ ND). If this is true, the *as-judged-by-themselves* principle can justify a nudge that replaces the status quo option ND with D. What remains to be established, however, is whether the inference D ≻_s_ ND → D ≻_t_ ND is itself justified. If there are no reasons to believe that people’s stated preferences differ from their true preferences, then it is. If there are good reasons to believe that their stated and true preferences may come apart, then it is not. In the latter case, it remains unclear which of the two options should be set as default if the *as-judged-by-themselves* principle is our guide.

An alternative explanation of the status quo bias is that people form their true preferences only at the time when they actively choose (Slovic 1995; Cohen 2013; Chater 2018). In this case, a decision-maker sticks with the status quo option to defer not the alignment of her actual choice with what she already prefers, but the formation of her true preference itself. If one sees that forming a true preference requires one to make complex personal value trade-offs (recall Alice from the earlier example), it may be tempting to fall back to the status quo option as a temporary way out of this burdensome task. In this case, the *as-judged-by-themselves* principle cannot justify a default rule nudge intervention because people’s true preferences are in actual fact incomplete (D ⊥_t_ ND) and the requirement R1 concerning people’s true preferences is not satisfied.

It may be possible to justify such intervention on other grounds. Instead of focusing on the *as-judged-by-themselves* principle, we may consider what would make people better off *by their own lights*.^3^ People may lack a preference over becoming and not becoming a potential organ donor (and they may avoid forming one) because of feeling queasy contemplating their end of life (Bovens 2009). Yet, if they hold a general preference to enhance other people’s well being, don’t subscribe to religious norms that would preclude them from donating organs, and do not care much about the integrity of their corpse for any other reasons, then their overall preference structure may dictate that they would form a preference to become an organ donor if it weren’t for their resistance to approach the issue on queasiness grounds (Bovens 1992). In this case, people’s stated preferences may be taken to suggest not what people’s true preferences are, but what they would be if people resolved themselves to form them. Similarly as before, it then remains to be established whether there are good reasons to believe that people’s stated preferences may differ from what their true preferences would be if they actually formed them. If there aren’t, a nudge-based intervention could be justified on grounds that it meets the *as-would-be-judged-by-themselves* principle. If there are, then it may not.

### The second hypothesis (H2)

The second explanation for why defaults work is the reference-point-dependence of people’s actual choices due to their psychological inclination to be loss-averse (Kahneman et al. 1991; Camerer et al. 2003; Johnson and Goldstein 2003; Davidai et al. 2012; Sunstein and Reisch 2013). A decision-maker is loss-averse if losses impact her welfare by a greater extent than do equal-sized gains (Kahneman and Tversky 1984). This can make defaults “sticky” reference points that loss-averse decision-makers are reluctant to abandon—a phenomenon that also manifests as what is known as the endowment effect (Kahneman et al. 1990).

Consider Bob who associates a greater value with retaining his organs after death when he is by default endowed with the option of retaining his organs (as it is the case in opt-in systems) than when he is not (the case in opt-out systems). If, as a result, the value that Bob associates with retaining his organs exceeds the value that he associates with becoming a donor in the former setting (opt-in) but not in the latter setting (opt-out), a simple cost–benefit evaluation of his options may lead Bob to stick with the default irrespective of what the default choice is.

A characteristic feature of loss-averse decision-makers is that the way they value their considered options depends on defaults, and there is some empirical evidence to support this in the context of organ donation. For example, Davidai et al. (2012) found that the way people construe their choice options in decisions concerning posthumous organ donation indeed varies with their reference point. In a set of surveys, participants in the USA, Germany, and Austria were asked to report how significant one’s decision to become or not to become a potential organ donor is compared to various other possible acts. Their responses varied significantly depending on whether they themselves lived in a country that operated under the opt-in (Germany) or the opt-out (Austria) system, or whether the people whose actions they were asked to consider lived in a country that operated under the opt-in (e.g., The Netherlands) or the opt-out (e.g., Belgium) system. For example, according to the results in one of the studies, “in an opt-in country organ donation is seen more like leaving 50% of one’s estate to charity than like leaving 5% and more like taking part in a political campaign than like voting for a mayor”, whereas “in an opt-out country, not agreeing to be a donor is seen as more like skipping your child’s graduation than like skipping your child’s baseball game and more like belittling someone who tried hard and failed than like not being supportive of someone who did so” (pp. 15202–15203). The fact that people perceive one’s decision to become a potential organ donor in an opt-in system as a significant prosocial act can be interpreted as suggesting that becoming a donor in the opt-in system is associated with bearing a significant personal cost. This would be the case if the value attached to retaining one’s organs is high. Similarly, the fact that people perceive one’s decision not to be a donor in an opt-out system as a significant antisocial act can be interpreted as suggesting that the value associated with retaining one’s organs in the opt-out system is low.

Although it is not outright clear what a loss-averse decision-maker’s latent (purified) preference is, this allows in principle to infer from people’s stated preferences that they truly prefer being potential organ donors (D ≻_s_ ND → D ≻_t_ ND) and to assume that, in an opt-in system, their actual choices are distorted by their psychological inclination to be loss-averse (ND ≻_r (opt-in)_ D despite D ≻_t_ ND). If this is true, the *as-judged-by-themselves* principle can justify a nudge that replaces the default option ND with D. As before, what remains to be established is whether the inference D ≻_s_ ND → D ≻_t_ ND is itself justified.

### The third hypothesis (H3)

The third explanation for why default rule nudges are effective is people’s perception of the default option as one that is implicitly recommended to them by policy-makers who are thought to be experts in the decision problem at hand (Johnson and Goldstein 2003; McKenzie et al. 2006; Halpern et al. 2007; Davidai et al. 2012; Sunstein and Reisch 2013). This accords two (mutually exclusive) assumptions about people’s true preferences. One is that people’s true preferences are incomplete (D ⊥_t_ ND) and that people align their actual choice with whatever they think is recommended to them (ND ≻_r (opt-in)_ D and D ≻_r (opt-out)_ ND). The other is that people’s true preferences are conditional on the preferences of the policy-maker: people truly prefer whatever option they think the policy-maker prefers and they take the recommended default option to be that.

We can interpret conditional preferences under the second assumption in two ways. On one interpretation, the policy-maker’s recommendation (i.e., the default option) sets the context in which choices are made and people’s true preferences are therefore context-dependent: ND ≻_t (opt-in)_ D and D ≻_t (opt-out)_ ND. On another interpretation, people’s true preferences are not over the options D and ND as such, but over whatever option people deem to be the recommended option to them (call it R) and the alternative (NR): R ≻_t_ NR. It is worth noting that since the standard theory of rational choice requires rational preferences to be stable and context-independent (see, e.g., Hausman 2012), the first interpretation raises the question of whether people’s true latent preferences, being context-dependent, are in fact rational. It is obvious that default rule nudges work only if people’s revealed preferences (that is, actual choices) are context-dependent. And a large body of empirical research has repeatedly shown this to be so: people’s choices reveal preferences that are shaped by seemingly irrelevant contextual market cues (Loomes et al. 2003, 2010; Tufano 2010; Isoni et al. 2016; Beraldo et al. 2019). However, for the *as-judged-by-themselves* principle to have a meaningful normative force in the practice of nudge, this cannot be so for people’s true latent preferences. For if they too are context-dependent and, as such, not rational, the whole idea that a nudge-based intervention can in principle align people’s actual choices with what their ‘better’ inner *rational* selves truly prefer is brought into question.

Be that as it may, if H3 is the correct explanation for why default options determine people’s actual choices, it is not possible to use the *as-judged-by-themselves* principle to justify a default rule nudge intervention. If people’s true preferences are incomplete (D ⊥_t_ ND), the requirement R1 is not satisfied. If people’s true preferences are conditional on the preferences of the policy-maker, people’s actual choices are tautologically aligned with their true antecedent preferences irrespective of what the pre-intervention default option is. In this case, a nudge-based intervention would fail to satisfy the requirement R3.

**Fig. 1:**
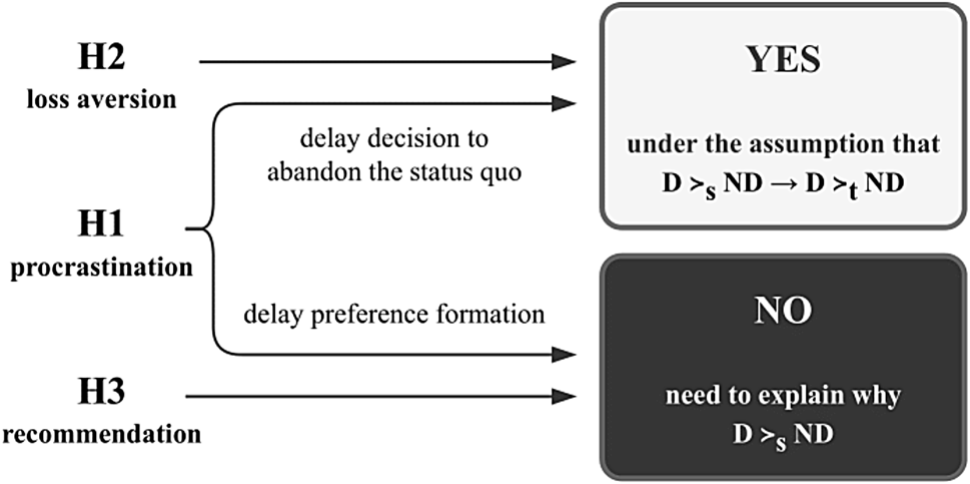
Does the *as-judged-by-themselves* principle justify a default rule nudge intervention that replaces the status quo option ND with D?

Figure 1 summarizes our argument. The hypotheses H1 and H2 allow in principle to make inferences about people’s true preferences that make it possible for the *as-judged-by-themselves* principle to justify a default rule nudge intervention. However, this is conditional on the assumption that people’s stated preferences are a reliable indicator of what their true preferences are, shown by the need to assume that the inference D ≻_s_ ND → D ≻_t_ ND is justified. The hypothesis H3 and the particular case of H1 that suggests people’s true preferences to be incomplete (D ⊥_t_ ND) do not make this possible. What needs to be explained in these latter cases is why people’s stated preferences D ≻_s_ ND (particularly, in an opt-in system) do not imply their true preferences D ≻_t_ ND. Note also that the hypotheses H1 and H2 in and of themselves are neutral with respect to whether D ≻_t_ ND or ND ≻_t_ D is the case. As such, a default rule nudge intervention can be justified on grounds that it meets the *as-judged-by-themselves* principle only if either H1 or H2 is the true explanation for why defaults work in conjunction with the inference D ≻_s_ ND → D ≻_t_ ND being justified too.

### Defaults as recommendations

McKenzie et al. (2006) conducted a set of laboratory-based experiments to investigate whether people (in this case, university students in the USA) perceived the default option as one that is implicitly recommended to them by policy-makers. In the study that specifically addressed posthumous organ donation, the majority of participants indeed thought this to be so. Although the scenario that the experimenters then used to investigate whether this also caused people to stick with the default option did not address organ donation, their analysis showed that perceived recommendations indeed played a causal role in determining participants’ actual choices.

Further support for this can be drawn from results of the statistical analysis carried out by Mossialos et al. (2008) of data from a cross-national survey that was conducted in 2002 spanning 15 European Union member states. The authors found that the survey respondents from countries that operated under the opt-out (presumed consent) system were more willing to donate their organs after their death than the respondents from countries that operated under the opt-in (explicit consent) system at the time. While correlation does not imply causation (in fact, when discussing their findings, the authors suggest it being possible that a country’s adopted organ donation policy reflected *prior* public attitudes in that country) we may expect this to be so if the hypothesis H3 were the true explanation for why default rule nudges work in this context. If people perceive the default option to be one that is recommended to them by policy-makers, they may also be more willing to choose that option than they would be if that option was not the one that they saw as being recommended to them.

### Are stated preferences true preferences?

We now consider whether there are reasons to suspect that the inference D ≻_s_ ND → D ≻_t_ ND that is needed to justify a default rule nudge intervention if the hypotheses H1 or H2 are true is not justified. The dichotomy between what people say they want to do and what they actually do has been studied by behavioural economists, who distinguish between people’s stated and revealed preferences, and psychologists, who refer to the phenomenon as the attitude-behaviour gap. Although our knowledge of what causes this gap in the context of posthumous organ donation is scant, a substantial amount of research has addressed this question in the study of people’s purchasing behaviour. Factors that have been identified as possible causes of the dichotomy in the latter context can shed some light on what we may expect in the case of organ donation.

Carrigan and Attalla (2001) survey evidence pointing to the persisting gap between consumers’ expressed socially responsible attitudes and their actual purchasing behaviours. For example, one study found that although the majority of consumers state that they would stop buying products of a particular brand if it came to be known that child labour was used in the respective company’s production process, significantly fewer of them translate this into actual action. Based on their own findings, Boulstridge and Carrigan suggest that “although consumers express willingness to make ethical purchases linked to good reputation, the reality is more likely to be that responsible corporate behaviour is not the most dominant criterion in their purchase decision. Price, quality and convenience are still the most important decision factors, with consumers purchasing for personal reasons rather than societal ones” (2000, p. 359).

Carrington et al. discuss evidence suggesting that the attitude-behaviour gap in people’s purchasing behaviour occurs partly due to contextual factors, e.g., “stand-out visibility [of choice options]”, “visuals to symbolically and effectively communicate the ethical credentials of the product” (2010, p. 155), that influence people’s choices via subconscious psychological mechanisms at point of purchase, and partly due to more stable conscious considerations, e.g., “extenuated time commitments and competing ethical demands” (p. 149). The authors argue that when people express their preferences verbally, they may “respond with answers they believe to be socially acceptable, overstating the importance of ethical considerations in their buying behaviour” (p. 141). This *social desirability bias* occurs “when people feel social pressure to respond with answers … that they believe to be socially acceptable” (p. 143). Papaoikonomou et al. discuss this as a serious limitation of survey-based methods aimed at eliciting people’s true preferential attitudes and warn that “consumers will give misleading answers and hide their true opinions on ethical purchase behavior” (2011, p. 81).

This suggests that people’s stated preferences may often reflect their *partial* and not *total* evaluative rankings of considered options. Because in surveys, when asked to express their preferences verbally or in writing, people do not actively choose, they may report a *partial* evaluative ranking that is indeed true, e.g., “yes, I am in favour of posthumous organ donation, generally speaking”. The all-things-considered clause of the requirement R2, however, will not hold if such stated preferences don’t reflect *all* reasons that people have for becoming or not becoming a potential organ donor.

Reviewing previous research and their own empirical findings, Papaoikonomou et al. (2011) identify a number of factors that inhibit people’s ethical purchasing behaviour, some of which can play a role in decisions concerning posthumous organ donation as well. For example, people may assume that their personal decisions don’t matter much because of the low overall social impact those decisions have. People may also face conflicts between their willingness to become potential donors and other social or familial obligations. For example, Bird and Harris (2010) discuss “worries about a “big brother” database state” as one factor that is responsible for people’s decisions not to become potential donors in countries that operate under the opt-in system. Also, 1 in 4 survey respondents in the European Union cited unease with posthumous manipulation of their bodies as their reason not to donate (European Commission 2010, p. 26).

Spain implemented an opt-out system in 1979, but its rate of actual posthumous organ donations—presently one of the highest in the world—started to increase only 10 years later (Matesanz and Domínguez-Gil 2019). The experts in Spain stress that their country’s success is attributed not to the adoption of the opt-out framework itself, but to a mixture of other policies that were implemented over the past decades (Matesanz et al. 2011, 2017). As Rodríguez-Arias et al. note, “Success factors of the Spanish Model include its legal approach and a comprehensive programme of education, communication, public relations, hospital reimbursement, and quality improvement” (2010, p. 1109). Some of these actions, in particular those aimed at “constructing a positive social climate toward donation and generating society’s trust in [the] system” (Matesanz et al. 2011, p. 335), can play an important role in shaping people’s all-things-considered *total* evaluative rankings of considered options. For example, Brazil adopted the opt-out framework in 1997 but later abandoned it because the switch was believed to have “aggravated [people’s] mistrust in the healthcare system” (English and Wright 2007, p. 1089; Csillag 1998; Ezaz and Lai 2019). This suggests that, when it comes to organ donation, people’s all-things-considered preferences can depend on considerations other than their general willingness to donate organs that they report in surveys. For example, someone in Brazil may be unwilling to become an organ donor because her all-things-considered judgement depends on the perceived inefficiency of the organ donation process and her trust in the healthcare system as a whole. As Dalal notes, “Unfortunately, many organs are buried rather than donated … because potential donors and their families believe that the organ distribution system is unfair and potential donors may receive less aggressive medical care” (2015, p. 46). Indeed, 1 in 5 people from those unwilling to become potential donors in the European Union cited distrust in the system as their reason not to donate (European Commission 2010, p. 26) and regarding people’s stated willingness to donate organs, Spain ranked only 13th and just above average among the European Union member states (p. 17).

## Expanding nationwide surveys

In light of the persisting gap between the supply and demand for organs in many countries, it is certainly good news if the *as-judged-by-themselves* principle can be used to justify a switch from an opt-in to an opt-out system when this, possibly combined with other policies, contributes to increasing the number of actual transplantations taking place. However, as we argued, it is often difficult to ascertain whether the principle can be used to justify this switch even in cases when nationwide surveys suggest that most people are generally willing to become donors. Whether the principle can be invoked depends on whether the requirements R1, R2, and R3 concerning people’s latent preferences are met as well as on which hypothesis (H1, H2, or H3) about the effectiveness of default rule interventions is most likely to be true.

Since it is likely that some people’s preferences will meet the requirements R1–R3 while other people’s won’t, and that each of the hypotheses H1–H3 may hold true for some people but not for others, we need accurate demographic knowledge of the proportion of people whose preferences meet these requirements and the extent to which each of the three hypotheses contributes to explaining the overall efficacy of default rule nudge interventions. It will be fruitful to expand future nationwide surveys by including questions that are specifically designed for that purpose.

For example, in order to uncover whether people already hold a preference over becoming and not becoming a potential organ donor, addressing R1, in addition to asking about their general willingness to donate organs, e.g., “Would you be willing to donate one of your organs to an organ donation service immediately after your death?” (European Commission 2010, p. 15), further questions could be added to find out if people have already formed an all-things-considered preference over the these options, e.g., “Have you personally already decided that you *will* or *will not* donate one or more of your organs to an organ donation service after your death if an opportunity to donate an organ arises?”. This could be immediately followed by questions aimed to uncover whether defaults are effective because people continually delay the formation of their preferences, addressing H1. For example, “If you personally have not yet decided on this matter, do you intend to decide on it later?” and “If you personally have not yet decided, have you postponed making a decision on this matter before?”.

In order to find out if default rule nudges work because of people’s tendency to procrastinate, we may ask “If you have personally decided that you *will* donate one or more organs after your death, but have not explicitly registered or otherwise stated your intent to do so, why is that?”. People could either be asked to freely state their reasons or be given a menu of options, e.g., “I am assumed to be a potential donor in virtue of the laws in my country.”, “I haven’t yet found the time to explicitly register or otherwise state my intent.”, “There are other reasons for why I have not explicitly registered or otherwise stated my intent.”.

In order to get a better understanding of the dichotomy between people’s stated and revealed preferences, addressing R2, a series of pairs of questions could be asked similar to these: “To what extent do you trust the organ procurement and distribution system in your country of residence?” and “Is the organ procurement and distribution system an important factor, and one that you in fact consider, when you make a decision on whether to become or not to become a potential organ donor?”. Similar pairs of questions could address (i) the fairness of the organ distribution system, (ii) worries that one would receive substandard care from healthcare professionals when hospitalized towards the end of life if known to be a potential donor, (iii) worries that one’s body will be unacceptably mutilated after death if one’s organs are to be used for transplantation, (iv) religious considerations, and so on.

Further questions could aim to uncover whether people’s preferences are conditional on what they believe policy-makers recommend to them, addressing R3, and whether this could also explain why defaults work, addressing H3. For example, having first established that people are informed about the organ procurement and donation policy in their country of residence, we could ask “Do you think that your country’s legislation recommends you to become a potential donor after your death?”, “Do you think that policy-makers in your country expect you to become a potential donor after your death?”, and “Are you willing to defer your decision on whether to become or not to become a potential donor after your death to expert policy-makers and to follow their recommendation on the matter?”.

## Mandated active choice

A mandated choice policy is an alternative to the use of the opt-in or the opt-out framework: instead of assuming one of the options to be one’s default choice, it requires everyone at some point (or at a number of points) during their lives to actively decide and choose. It is certainly a coercive policy and the question of when people ought to decide on posthumous treatment of their organs is not easy to answer. Nevertheless, it has been discussed as a viable way to overcome a number of problems associated with default rule nudging, some of which we discussed in this paper (Thaler and Sunstein 2008; Hansen 2012; Cohen 2013; MacKay and Robinson 2016; Wilkinson and Wilkinson 2016). Here we want to offer four reasons for why mandated active choice is better than the opt-in and the opt-out systems if upholding the *as-judged-by-themselves* principle is indeed a policy-maker’s main objective.

MacKay and Robinson (2016) discuss the potential difficulty of making a decision on the posthumous treatment of one’s organs to be one of the drawbacks of forced choice. Forcing people to make a difficult decision is coercive and unattractive. However, it is interesting to consider the difficulty of making a decision in this context from the point of view of the existence (or not) of people’s actual all-things-considered preferences over the options at hand. If, as a matter of fact, people have an all-things-considered preference over becoming and not becoming a potential donor but fail to satisfy that preference due to, for example, their tendency to procrastinate (one case when a switch from an opt-in to an opt-out system can be justified on the basis of the *as-judged-by-themselves* principle), then mandated active choice should not be problematic. After all, if one already has an all-things-considered preference to become or not to become an organ donor, a choice, whether it is forced or not, should be easy to make. If, on the other hand, people struggle to make up their minds when requested to do so, this would suggest that they do not in fact hold an all-things-considered preference over the considered options to begin with, i.e., their preferences are incomplete. But if their preferences are incomplete, mandated active choice is better suited than default rule nudging to ensure that the *as-judged-by-themselves* principle will in the end hold. The reason being is that it is the decision-maker herself who is ultimately in the best position to work out what she wants. If the decision-maker forms her preference when she actively chooses, forced choice would require her to form a preference even if she does not have one to begin with.

Second, a switch from an opt-in to an opt-out system is sometimes justified using the *fewer mistakes argument* (Prabhu 2019). The argument begins with the assumption that the majority of people truly want to become potential organ donors (based, for example, on people’s stated preferences expressed in nationwide surveys). Irrespective of the actual cause of the default rule’s effectiveness, a policy-maker will thus commit fewer mistakes—from the point of view of ensuring the satisfaction of people’s all-things-considered preferences—under the opt-out system. However, as Zambrano argues in an unpublished article (titled “Fewer mistakes and presumed consent”), mandated active choice is in an even better position to minimize the number of mistakes made if the *as-judged-by-themselves* principle and the satisfaction of people’s all-things-considered true preferences are the policy-maker’s primary goals (see also Hansen 2012). Although adopting the opt-out system may indeed result in fewer mistakes committed in comparison to the opt-in system, mandated active choice would result in no (or close to no) mistakes, which is even better.

The third reason why mandated active choice may be more appropriate than a default rule nudge is related to the fact that families of deceased persons often decide not to donate their deceased relatives’ organs precisely because their true preferences are unknown (Thaler and Sunstein 2008; Wilkinson and Wilkinson 2016). The fact that most people never discuss the issue of post-mortem organ donation with their families gives good reason to believe that many deceased persons did not have well-formed stable preferences over the options before they died. If true, then the requirement R1 may not hold for a large portion of the population and, as such, a mandated choice policy may indeed be better than a switch to opt-out.

Lastly, many choices relating our healthcare are already mandated. In many countries, everyone is required to take out personal health insurance. It is also mandatory for drivers to hold insurance that protects third parties. In this light, a mandated choice policy in the context of posthumous organ donation should not be that alarming. Of course, a personal health insurance plan is meant to benefit the person who holds it and a driver’s car insurance is required to compensate those to whom the driver may actively cause harm. Posthumous organ donation in many respects differs from these scenarios. However, in the two former cases, we often have no choice at all. In the latter, a mandated choice policy would simply require us to make a choice. Indeed, if our ultimate goal had nothing to do with autonomous choosing and was simply to maximize the number of actual transplantations performed, forcing everyone to donate their organs after death would be the most effective policy (Thaysen and Albertsen 2020).

## Conclusion

We analysed the most common explanations for the effectiveness of default rule nudge interventions in the context of posthumous organ donation and examined whether the hypothesized explanations of the default rule’s effectiveness accord us the possibility to justify a switch from an opt-in (explicit consent) to an opt-out (presumed consent) system on the basis of the *as-judged-by-themselves* principle. We found that only under specific circumstances—cases in which there are good grounds to assume the existence of decision-makers’ all-things-considered true latent preferences, combined with people’s tendency to procrastinate or to be loss-averse—we can use the *as-judged-by-themselves* principle to justify the switch. In other cases caution is needed if the principle is meant to guide public policy.

This is not to say that nudging people into becoming potential organ donors cannot be justified on other grounds. However, if people are not nudged into choosing what they themselves deem to be their most preferred option, i.e., if the *as-judged-by-themselves* principle does not apply, nudging may require more detailed public debates that involve the very people who are targeted by nudge. This may be particularly important if public authorities want to avoid the accusation that nudging is manipulative and thus prevent resistance to nudging in other domains. Such resistance would certainly be lamentable in cases in which the *as-judged-by-themselves* principle is actually met.

Lastly, we offered four reasons for why mandated choice policy may be better than default rule nudging when it comes to posthumous organ donation and, in this light, our position coincides with that of Thaler and Sunstein in their original take on this question (2008, p. 180). In our daily lives, there are many noble things we know we should do but never do them. All too often, life gets in the way. Thinking about and deciding what should happen to our organs after we die may be one of those things. And making that decision can also feel queasy. Setting an official deadline in our busy lives to do it might not be such a bad thing after all.

## Data Availability

Not applicable.

## Footnotes

1 New Zealand, the United Kingdom, and several states in the USA have used some form of mandated choice policy (Hansen 2012; Thaysen and Albertsen 2020). Not everyone, however, may be forced to choose. In New Zealand and the United Kingdom, for example, people are only required to state their choice upon receiving or renewing their driver’s license.

2 This should include cases where people have a preference not only over ends (e.g., being a donor versus being a non-donor) but also over means through which those ends are attained (Sunstein 2016, p. 55). In other words, people must strictly prefer the option which they are nudged into choosing to the option which they choose in the absence of the nudge *in the respective contexts* in which they make the two choices (e.g., they must strictly prefer *being a donor in the opt-out system* to *being a non-donor in the opt-in system*).

3 We thank Luc Bovens for this point and the example that we will use to illustrate it.
